# Treatment of Cystic Craniopharyngioma with Intracystic Stereotactic Instillation of Phosphorus 32

**Published:** 2017

**Authors:** Sohrab Shahzadi, Ahmad Soltani, Andia Shahzadi, Khosrow Parsa

**Affiliations:** 1 Shohada Hospital, Shahid Beheshti University of Medical Sciences, Tehran, Iran; 2 Department of Neurosurgery, Shiraz University of Medical Sciences, Shiraz, Iran; 3 University of Toronto, Toronto Canada; 4 Iranmehr Hospital, Tehran, Iran

**Keywords:** Cystic craniopharyngioma, Stereotactic, Phosphorus 32, Treatment

## Abstract

**Objective:**

Cystic craniopharyngiomas are considered the most common intracranial nonglial tumor in children with the tendency for cyst formations. The aim of this study was to evaluate the effect of intracystic phosphorus 32 (P32) therapies on controlling the growth of the cystic component of craniopharyngioma.

**Materials & Methods:**

This clinical study was conducted on 47 patients with cystic craniopharyngioma from March 1998 to June 2012 at Shohada Tajrish Hospital, Tehran, Iran.

Patients were treated with stereotactic intracystic P32. The mean cyst volume was 23.5 ml, and the dose of radiation to the inner cyst wall was 250 Gy.

**Results:**

The overall response rate was 78.1% and the mean survival was 113.1±11months.

The survival rate at 1, 3, 5, and 10 years after p32 therapy was 91%, 77%, 73%, and 52%, respectively. There was no mortality related to the procedure and no visual or endocrinal deterioration. Visual improvement occurred in 88% of patients presented with recent deterioration due to the cyst enlargement.

**Conclusion:**

Intracystic p32 therapy was an effective and almost safe procedure for the treatment of cystic component of craniopharyngioma.

## Introduction

tumor originating from the embryonic epithelial cells of the craniopharyngeal duct, or from the metaplasia of the squamous epithelial cell. It is considered the most common intracranial non-glial tumor in children (1). Craniopharyngioma is composed of 1.2%–4% of the primary intracranial neoplasms and accounts for 5%– 10% of intracranial tumors in children (2, 3).

Although pathologically it has a benign appearance corresponding to WHO grade ӏ, the clinical behavior is aggressive causing morbidity by damaging the hypothalamus, pituitary gland, and optic chiasm (4, 5). Craniopharyngioma has variable appearances and presentations; therefore, treatment of each case should be individualized (2). 

Surgical resection via microsurgery or endoscopic approaches is particularly for tumors with large solid part. There are high mortality and morbidity in open surgery (6). Even after total resection, recurrence rate after 10 years is around 18% (6, 7).

Cysts of the craniopharyngioma usually adhere to the critical surrounding neural and vascular structures and are frequently considered inoperable (8). Accordingly, less aggressive surgery along with adjuvant therapy such as external radiation and intracystic therapy can comparatively provide the control of tumor with less morbidity than aggressive surgery (4, 8).

Intracystic therapy using substances including β emitting radioisotopes (phosphousr32 (P32), yttrium90, aurum198, and rhenium186) destroys the epithelial lining of the cyst wall and suppresses the re-accumulation of the cyst (5, 9, 10). Intracystic treatment provides durable cyst shrinkage and volume reduction by simply repeated aspiration (3, 5). Nonetheless, it is not effective on the solid part of the tumor. 

The aim of this study was to determine the treatment results for patients with cystic craniopharyngioma after intracystic instillation of P32 radioisotope. 

## Material & Methods

This clinical study was conducted on 47 patients with cystic craniopharyngioma from March 1998 to June 2012 at Shohada Tajrish Hospital, Tehran, Iran. 

Previously, in the same treatment center, P32 therapy was used on 22 patients with shorter duration of followup from 1998 to 2005 (1). The current study focused on the outcome of 47 patients with longer follow-up period from 1998 to 2012. Six patients were excluded from this study due to their unwillingness. Data were collected from the patients’ medical records regarding presenting symptom, neurological and ophthalmological examinations, previous therapies, endocrinological tests, imaging, and definite pathology. 

The most common presenting symptoms were headache and poor vision. Other presenting symptoms included ocular deviation, hemiparesis, cognitive changes decline, and hearing problems. Fourteen patients (34.1%) presented with progressive cyst enlargement during the routine follow-up without any significant symptoms. Endocrinopathy was detected in 87.7% and five patients had normal endocrinal profiles. Patients had undergone treatments before P32, which included craniotomy (39 patients), external beam radiation (36 patients), ventriculoperitoneal (VP) shunt (17 patients), gamma knife therapy (7 patients), stereotactic biopsy (one patient), and transsphenoid surgery (TSS) (one patient).

The indication of P32 therapy was a documented cyst enlargement after unsuccessful prior therapies and when the diagnosis was first made by a stereotactic biopsy of a suprasellar cystic mass. The patients were divided into three groups according to brain CT scan or MRI before P32 intracystic therapy: 1) solitary cystic, 2) multicystic: with more than one cyst and small solid part, and 3) mixed solid and cystic. For delivery of P32 the stereotactic system, Riecherd-mundinger was used if the omayo reservoir had not been implanted before or if it was not functional. The head was fixed in Riecherdmundinger under a local anesthesia and intravenous sedation without intubation. Frontal burr hole was placed and the cyst punctured by a cannula with its sharp tip. After aspiration of the large cysts and reassurance of the target, ommaya or similar shunt was placed into the cyst. Contrast material was injected via the reservoir of ommaya and X-Ray taken to exclude any leakage from the cyst. The cyst volume was measured using the vepro image processor. The amount of P32 that deliver 250 GY to the cyst wall for about five-half-life (around 70 d) was calculated. With safety routine precautions in handling the radioactive substance, the determined dose of P32 was delivered via the catheter. Then, all the radioactive contaminated materials were handled with routine care and disposed of. A neurosurgeon evaluated the patients neurologically and after 48 hours monitored them by a radioactive detector. Ideally, there should be no systemic exposure to radioactivity so that the only radioactive detected in the cyst and in the ommaya, but not in the liver. The patients were discharged from the hospital and followed up every week for a month, then every three months for one year, and after that each year. 

All patients were examined with CT scan, endocrinal lab data, and ophthalmological examination.

The treatment response was defined according to the changes, which occurred in the cyst size in the CT scan examination in four statuses as follows: 

1) Complete response, when the cyst disappeared

2) Partial response, when the cyst size decreased, but was not disappeared

3) Stable, when no changes were seen in the cyst size

4) Progressive, when the cyst size was enlarged

Statistical analysis was performed using SPSS 17 (Chicago, IL, USA) and survival rate was evaluated by Kaplan-Meier curve to describe the duration of the survival from the time of the injection of P32 to the end of the study or the patient’s death.

## Results

Forty-one patients (61% men) with an age range of 4 to 54 yr (mean age of 17.2±11.3 yr) were enrolled. The mean cyst volume was 23.5 ml (range from 4.5 ml to 80 ml). Nine patients (22%) showed complete response to P32 therapy and cyst disappeared. In addition, 23 patients (56.1%) showed partial response and cyst decreased in size, so overall response rate including complete response and partial response was 78.1%. 

**Fig 1:**
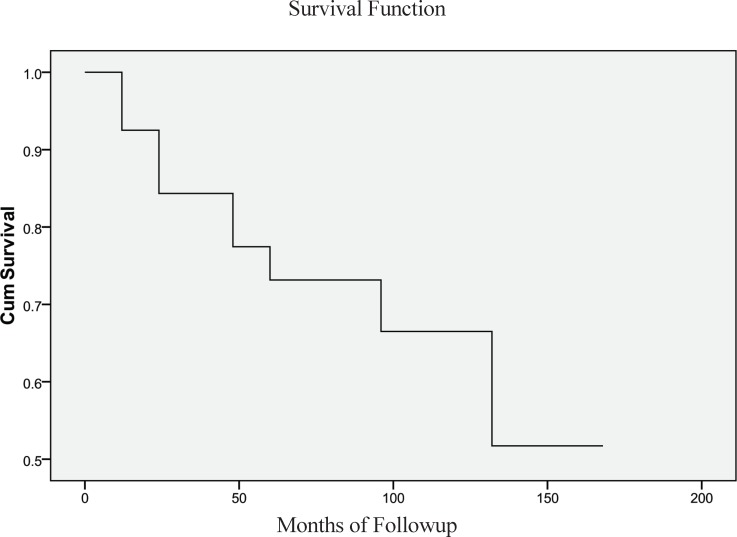
Survival Function

Seven (17%) patients showed no change in the cyst size while two (4.8%) patients showed an increase in the cyst size. Overall, 17 patients (41.46%) required no further treatment after one session of P 32 therapy and among them six showed complete response, and 11 showed partial response. In the responsive group, six patients had drainage and 12 needed to receive further courses of therapy (10 patients had two, one patient had three, and one patient had four courses of treatment). The mean follow-up duration of patients was 58.02 months (7-158 months). The 1, 3, 5, and 10-year survival was 91%, 77%, 73%, and 52%, respectively. Using Kaplan-Meier survival analysis, mean survival was 113.1 ±11months (95% CI, 91.5 -134.6) (Figure 1). Out of patients with craniopharyngioma, seven died due to tumor progression, three due to causes other than enlargement of the cyst and one due to enlargement of other cysts in the tumor did not receive P32 therapy, in spite of observing the good treatment response in the cyst that received P32 therapy.

Prior to the treatment, 39 patients (95.1%) had impaired vision. After P32 therapy, 18 patients (43.9%) showed improvement in their vision, 21 (51.2%) had unchanged vision, and two (4.9%) who had normal vision prior to therapy remained normal. Therefore, there was no new visual impairment after the therapy. Among the 39 patients with decreased vision, 22 patients (53.6%) had a recent visual deterioration because of the cyst enlargement whereas, after P32 therapy, 18 of them (81.8%) showed visual improvement.

Overall, 36 patients had endocrinopathy before the therapy with P32, and after that there were no changes in the endocrine profiles. All the patients were monitored by radioactive tracer around the head and liver for detection of any leakage from the cyst within the first 48 hours. 

Accordingly, no patient showed radioactive detection in the liver that means there was no radioisotope leakage from the tumor cyst. 

Complications related to the therapy were few and included one temporally CSF leakage from the scalp wound, one brain abscess, one superficial wound infection, and one temporal sixth cranial nerve palsy. There was no post-operative mortality.

## Discussion

There is no uniform protocol for the treatment of the craniopharyngioma. However, therapy should minimize the risk of complications and should be individualized for any patient (2). Surgery (transcranial and endoscopic) is often the first choice treatment (6, 7, 11). Radical resection, if feasible, offers the best chance for disease control and possibility of cure (12). However, it is a challenge, with possible complications including visual and vascular injuries, endocrinal hypothalamic damage, obesity, and behavioral changes (13-15). Repeated surgery has a high failure rate (16), and the outcome of surgery depends on the expertise of the surgeon (5). Gross tumor removal has been reported around 45%-85% with a 5-year recurrence free survival around 76%-86.9%, and after partial tumor removal a 5-yr recurrence-free survival about 40%- 41.7% (6, 11). After partial resection with radiotherapy there were 5 yr recurrence-free survival rate about 90% and 10 yr overall survival rate more than 90% (3, 4, 15). The cysts of craniopharyngioma are difficult to remove completely because of the attachments to the surrounding vital structures (8); therefore, repeated aspirations are needed (3, 5). Intracystic therapies include instillation of radioactive substances (bleomycin and interferon) which destroys the secretary epithelial lining of the cyst. These therapies lead to shrinkage of the cyst, providing a good local control and reducing the need for repeated aspiration and further surgeries (3, 5). Intracystic β emitting radioisotopes include P32, aurum198, rhenium186, and yttrium90 (5, 8, 9). 

Although there is no overall difference clinically among isotopes, some authors are in favor of P32 because of a mean β energy release of 0.69MeV, longer half-life (14.3 d), and less tissue penetration (2–8 mm). The latter item reduces the risk of damage to the adjacent structures including visual pathway (1, 5). P32 is a highly effective and safe treatment and may be the first and only therapy required in solitary cystic craniopharyngioma for a long time (17, 18).

In our study, intracystic P32 instillation led to tumor response rate of 78.1%, comparable to previous studies with response rate of 67%-88% (5, 10, 17-19). Moreover, 5 and 10-yr survival in our research was 73% and 52%, respectively; this is consistent with some studies (20, 21) and slightly lower than the other studies (22). Generally, these differences in the survival rate can be attributed to the fact that there are multiple variables affecting the survival in the patients. It is not uniform in all of the studies. The visual improvement has routinely been reported in most patients in different studies (19, 21, 23-25). In our study, visual improvement was 43.9%, which is also comparable with prior studies (19, 21, 24, 25). There was no visual decline after the procedure, but several studies showed visual deterioration after the therapy (21, 25, 26). The possible causes of visual deterioration (26) were the increase in the size of the solid part, new cyst formation, and failure of the cyst collapse. The presence of optic atrophy before the brachytherapy had a remarkable association with the visual deterioration. In the present study, the major determinant of improvement of the visual function was a recent deterioration of vision due to the cyst enlargement. It included 22 out of all 39 patients with visual defects. After therapy with P32, vision improved overall in 18 patients (43.9%); that is, 81.8% of the patients with recent deterioration showed amelioration of visual function. 

Regarding systemic leakage of P32 from the cyst, gamma scan 48 h after the therapy showed no cyst leakage, but some studies reported 10% leakage after instillation of P32 (20).

It will be helpful to investigate the change in the efficacy of intracystic P32 therapy after a period of serial drainage of the cyst, aiming to reduce the total volume of the cyst before P32 therapy. It also contributes to the reduction of the total dose of P32, so reducing the risk of its side effect. Considering different pathologies of craniopharyngioma (adamantinomatous and squamous papillary types), comparing the effect of P32 in cystic craniopharyngiomas according to their specific pathology may have prognostic value. 

**In Conclusion, **cystic craniopharyngioma treatment with intracystic p32 was a safe therapy for the cystic component of craniopharyngioma. It might be a good choice for a long duration of time in purely cystic craniopharyngioma and could eliminate or reduce the need for surgery or repeated aspiration. However, P32 therapy cannot prevent the growth of the solid part or appearance of new cysts.
